# Gut Microbial Dysbiosis Is Associated With Profibrotic Factors in Liver Fibrosis Mice

**DOI:** 10.3389/fcimb.2020.00018

**Published:** 2020-01-31

**Authors:** Sizhe Wan, Yuan Nie, Yue Zhang, Chenkai Huang, Xuan Zhu

**Affiliations:** Department of Gastroenterology, The First Affiliated Hospital of Nanchang University, Nanchang, China

**Keywords:** liver fibrosis, NOX4, RhoA, microbiota, high-throughput

## Abstract

**Background and Aims:** Continuous development will evolve into end-stage liver disease. Profibrotic factors NOX4 and RhoA participate in the activation of HSC and accelerate the development of liver fibrosis. Abnormal intrahepatic metabolism during liver fibrosis interferes with intestinal homeostasis through the liver—gut axis.

**Methods:** Wild-type (WT), NOX4 knockout, RhoA expression inhibition C57BL/6 mice were randomly divided into 6 groups as follows: control group, CCl_4_ group, NOX4^−/−^ group, AP group, RhoAi group, and FA group.

**Results:** The results of alpha-diversity suggest that the diversity and abundance of intestinal flora in liver fibrosis mice is lower than that in normal mice, but there is some recovery in liver fibrosis mice with NOX4 or RhoA intervention. The flora structure showed that the intestinal flora of the control group, NOX4^−/−^ group, AP group, RhoAi group, and FA group belonged to one type, while the intestinal flora of the CCl_4_ group belonged to another type. In addition, analysis of the composition of the flora at the level of the phylum and genus also suggested the decline in Firmicutes and *Lactobacillus* caused by liver fibrosis has partially restore in the liver fibrosis mice with NOX4 or RhoA intervention. In terms of functional prediction, the “Secondary metabolites biosynthesis, transport and catabolism,” “Infectious diseases,” and “Xenobiotics biodegradation and metabolism” signaling pathways are mainly enriched in liver fibrosis mice, and the “Energy production and conversion,” “Defense mechanisms,” and “Carbohydrate metabolism” signaling pathways are mainly enriched in the NOX4 and RhoA intervention groups.

**Conclusion:** In the case of liver fibrosis, the intestinal flora is disordered, and the disorder is related to NOX4 and RhoA. This study provides theoretical support for a better understanding of the underlying mechanisms of liver fibrosis development.

## Introduction

Liver fibrosis is the result of liver damage repair caused by various damage stimuli, such as viruses, alcohol, schistosomes, and drugs (Lee et al., 2015; Seki and Brenner, 2015). Continuous development can lead to cirrhosis and even liver cancer (Tsochatzis et al., 2014). Once end-stage liver disease is reached, there is a lack of effective treatments today. As an early pathological change in chronic liver disease, liver fibrosis has received extensive attention due to its reversible characteristics. The conversion of hepatic stellate cells (HSCs) to proliferating myofibroblasts (MFBs) is a central event in the pathogenesis of hepatic fibrosis (Lee et al., 2015).

NADPH oxidase (NOX) is a multi-subunit transmembrane enzyme complex composed of seven members: NOX1, NOX2, NOX3, NOX4, NOX5 and two dual oxidases, DUOX1 and DUOX2. The subunits of each member are slightly different, and the activities of NOX4 are mainly regulated by NOX4, p22phox, and Poldip2 (Crosas-Molist and Fabregat, 2015). NOX4 participates in the regulation of signal transduction in HSCs by producing reactive oxygen species (ROS) (Liang et al., 2016). More than 20 members have been found in the Rho GTPase superfamily, of which RhoA, Rac1, and Cdc42 are the most studied Rho GTPases (Ridley, 2012). Rho GTPase is a key molecule in multiple signal transduction pathways in cells and can be involved in the actin framework, cell polarity, and gene transcription (Etienne-Manneville and Hall, 2002; Hanna and El-Sibai, 2013). RhoA and its downstream signaling molecules can be expressed on the surface of hepatic vascular smooth muscle cells, vascular endothelial cells and HSCs (Hennenberg et al., 2006), which can aggravate liver fibrosis by regulating HSC activation, migration, adhesion, contraction, proliferation, and apoptosis (Li et al., 2012; Hu and Phan, 2013). Both NOX4 and RhoA are profibrotic factors that play an important role in liver fibrosis, and they have a close interaction in fibrotic diseases (Manickam et al., 2014; Meng et al., 2015).

The intestine is an organ that is closely related to the liver in terms of anatomy and function; that is, 75% of the blood in the portal vein of the liver is supplied by the intestine (Henao-Mejia et al., 2013). Because of the presence of the liver-gut axis, the liver and intestine can interact with each other through a variety of physiological activities. Therefore, pathophysiological changes during liver fibrosis, such as changes in the immune response, inflammatory response, and bile acid metabolism, can affect intestinal homeostasis (Palma et al., 2007; Assimakopoulos et al., 2012).

In the healthy human body, the gene quantity of intestinal microbiota is ~3.3 million, with 500–1,500 species, mainly including 9 phyla, among which Bacteroides have the highest abundance, Firmicutes are the next, while Proteobacteria have low abundance (Lozupone et al., 2012). Its composition and diversity are greatly affected by factors such as individual differences, diet, and age (Kurilshikov et al., 2017; Llewellyn et al., 2018). The intestinal microbiota has been shown to be involved in a variety of life activities, such as energy metabolism, immune regulation, and inflammation, which are important for maintaining human health (Blander et al., 2017; Kootte et al., 2017; Jia et al., 2018). A large number of recent studies have confirmed that the intestinal microbiota are closely related to the development of many diseases (Byrd et al., 2017; Jie et al., 2017; Ni et al., 2017).

Intestinal microbiota disorders are often accompanied by chronic liver disease. The dysregulation of the microbiota has been confirmed in the study of cirrhosis, i.e., the decline of beneficial bacteria and the rise of harmful microbiota (Qin et al., 2014). The disordered intestinal microbiota enter the blood through the damaged intestinal barrier and is transferred to the liver, whereby immune responses and proinflammatory cytokines are induced by microbiota-derived pathogen-associated molecular patterns (PAMPs), which in turn aggravates the original liver disease (Kumar et al., 2011; Giannelli et al., 2014). However, the changes in the intestinal microbiota during liver fibrosis are not clear. We attempted to identify changes in the intestinal microbiota based on high-throughput sequencing techniques using a mouse model of liver fibrosis and to determine whether these changes have a correlation with the profibrotic factors NOX4 and RhoA.

## Methods

### Animal Models and Experimental Design

All wild-type (WT) C57BL/6 mice used in experiments were purchased from the Department of Laboratory Animal Science of Nanchang University, and NOX4 knockout C57BL/6 mice were purchased from the American Jackson Laboratory. The mice were kept in a specific pathogen-free (SPF) environment with a 12:12 h light/dark cycle with free access to sterile food consisted of beans and grains, and water, using corn cobs as bedding. C57BL/6 mice, aged 6–8 weeks, weighing 20–30 g were randomly divided into a control group, in which mice were gavaged with olive oil (2 ml/kg) twice a week for 8 weeks (*n* = 8); a CCl_4_ group, in which mice were gavaged with carbon tetrachloride (CCl_4_) (20% olive oil dilution, 2 ml/kg) twice a week for 8 weeks (*n* = 8); an NOX4^−/−^ group, in which NOX4 knockout mice were gavaged with CCl_4_ dissolved in olive oil twice a week for 8 weeks (*n* = 8); an AP group, in which, after gavage with CCl_4_ twice a week for 4 weeks, mice were gavaged with apocynin (AP) (40 mg/kg/d) and CCl_4_ at the same time for the last 4 weeks (*n* = 8); an RhoAi group, in which mice received adeno-associated virus (AAV) via tail vein injection for 1 week to inhibit RhoA (Supplementary Figure 1) and then were gavaged with CCl_4_ twice a week for 8 weeks; and an FA group, in which, after gavage with CCl_4_ twice a week for 4 weeks, mice were gavaged with fasudil (FA) (10 mg/kg/d) and CCl_4_ at the same time for the last 4 weeks (*n* = 8). All experimental procedures were endorsed by the Animal Care and Use Committee of Nanchang University and comply with the National Institutes of Health Guide for the Care and Use of Laboratory Animals.

### Liver Histology Analysis

Mouse liver tissue was fixed, dehydrated, and paraffin embedded. The embedded liver tissue was cut into 5 μm sections for haematoxylin and eosin (H&E) and Masson's trichrome staining. We randomly selected five fields of view for observation and evaluated liver damage and liver fibrosis based on METAVIR scoring criteria (Poynard et al., 1997; Table 1).

**Table 1:** METAVIR scoring criteria.

**Fibrosis grade**	**Comment**
F0	No fibrosis.
F1	The fiber area of the portal area is enlarged, but no septa are formed.
F2	The fiber area of the portal area is enlarged, and a few septa are formed.
F3	Numerous septa are formed without cirrhosis.
F4	Cirrhosis.

*F, Fibrosis*.

### Blood Indicator Test

An automatic biochemical analyzer was used to analyze the blood collected from mice and to detect the levels of ALT, AST, and TBIL in the blood (Department of Clinical Laboratory, First Affiliated Hospital of Nanchang University, China).

### Construction and Injection of Adeno-Associated Virus (AAV) for RhoA Inhibition

We injected the AAV type 9 expressing a short hairpin RNA (shRNA) directed at RhoA (AAV-shRhoA) into the mouse through the tail vein for RhoA expression inhibition.

The titer of the final AAV-shRhoA virus was 3.5 × 10^12^ viral particles/ml in phosphate-buffered saline (PBS). The plasmid was designed with GFP to serve as a carrier for shRNA.

### DNA Extraction and Sequencing

Upon mouse sacrifice, we collected anal fecal samples and immediately placed them in liquid nitrogen. Microbial DNA was extracted from the fecal samples using a stool DNA kit (Omega, China) according to the manufacturer's protocol. We designed and used primers 338F 5′-ACTCCTACGGGAGGCAGCAG-3′ and 806R 5′- GGACTACHVGGGTWTCTAAT-3′ to amplify the bacterial V3-V4 region of the 16S rRNA gene. Polymerase chain reaction (PCR) amplification was conducted using a commercial kit under the following conditions: 95°C for 3 min; 25 cycles at 95°C for 30 s, 55°C for 30 s, and 72°C for 30 s; and a final extension at 72°C for 10 min. The PCR product was extracted from 2% agarose gel and purified using the AxyPrep DNA Gel Extraction Kit (Axygen Biosciences, Union City, CA, USA) according to manufacturer's instructions and quantified using Quantus™ Fluorometer (Promega, USA). Purified amplicons were pooled in equimolar and paired-end sequenced (2 × 300) on an Illumina MiSeq platform (Illumina, USA) according to the manufacturer's guidelines. The raw 16S rRNA gene sequencing reads were demultiplexed, quality-filtered by Trimmomatic and merged by FLASH. All raw reads were deposited into the open resource database.

### Microbiota 16S Function Prediction

The 16S function prediction is to standardize the OTU abundance table by Phylogenetic Investigation of Communities by Reconstruction of Unobserved States (PICRUSt) (the PICRUSt software stores the Cluster of Orthologous Groups (COG) information and Kyoto Encyclopedia of Genes and Genomes (KEGG) information corresponding to the greengene id), that is, to remove the influence of the number of copies of the 16S marker gene in the species genome and then to correspond to each OTU. The greengene id obtains the COG family information and KEGG Ortholog (KO) information corresponding to the OTU. The abundance and KO abundance of each COG were calculated. According to the information of the COG database, the descriptive information of each COG and its functional information can be obtained, thereby obtaining the functional abundance spectrum; according to the information in the KEGG database, KO and Pathway information can be obtained, and the abundance of each functional category can be calculated according to the OTU abundance. The COG database is composed of functional classifications from the original, as well as functional annotations based on taxonomy. The KEGG database collects a series of functional units for genome annotation and biological interpretation.

### Quantitative Real-Time RT-PCR (qRT-PCR)

Liver tissue was homogenized in 1 ml of TRIzol (Life Technologies, USA), and total RNA was extracted. The integrity of RNA was verified via agarose gel electrophoresis, and RNA was converted to cDNA using a FastQuant RT kit (TIANGEN, cat. KR106-02, China). For qRT-PCR, SuperReal PreMix Plus (TIANGEN, cat. SYBR Green, China) was used to determine the quantitative expression of RNA under the following conditions: 95°C for 15 min, followed by 40 cycles of 95°C for 10 s and 60°C for 30 s. The number of amplification cycles was 41. GAPDH was the reference gene. The mRNA levels of RhoA were normalized to GAPDH mRNA levels. The qRT-PCR primers are shown in Supplementary Table 1.

### Statistical Analysis

We used SPSS 23.0 software for data analysis. GraphPad Prism 7.0 software was used for image production and output. Each experiment was repeated 3 times to ensure confidence in the results. A one-way analysis of variance (one-way ANOVA), Student's *t*-test, or the Mann-Whitney rank sum test was used to analyze the significant differences between groups. Means were considered different at *P* < 0.05.

### Profibrotic Factors NOX4 and RhoA Are Associated With CCl_4_-Induced Liver Fibrosis

**Figure 1:**
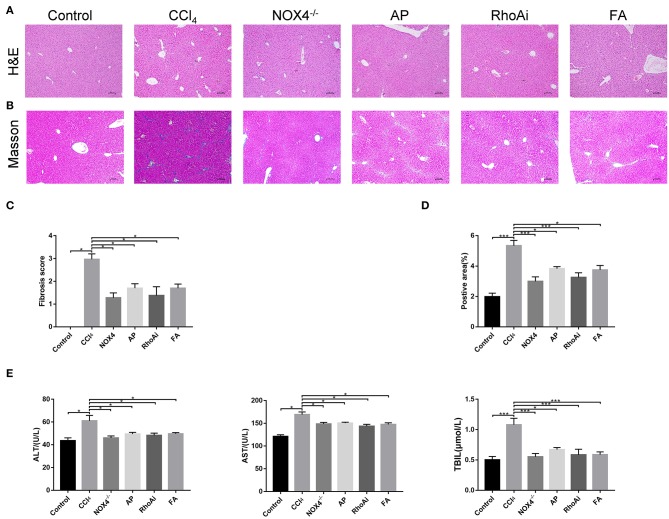
Effect of UA on liver injury and fibrosis. **(A,B)** Haematoxylin-eosin (H&E) staining and Masson's trichrome staining (100×) were used to assess liver damage and liver fibrosis. **(C)** Morphological statistics for liver fibrosis score in images. **(D)** Quantitative analysis of the area of fibrosis in the image. **(E)** Liver function-related serum indicators were detected. CCl_4_-induced hepatic fibrosis was relieved after intervention in the expression of NOX4 or RhoA. Data represent the mean ± SD of values for each group (*n* = 6). Student's *t*-test was used to analyze the differences between groups. *P < 0.05 and ***P < 0.001. CCl_4_, carbon tetrachloride; NOX4^−/−^, NOX4 knockout; AP, apocynin; RhoAi, RhoA inhibitor; FA, fasudil.

We verified the liver fibrosis-related mouse model. H&E staining showed increased inflammatory cell infiltration and hepatocellular necrosis in the liver of mice in the CCl_4_ group compared to the control group. However, in NOX4^−/−^ and FA groups, the inflammatory infiltration and hepatocellular necrosis of the liver showed a significant improvement. In the RhoAi and FA groups, inflammatory infiltration and hepatocellular necrosis also showed different degrees of decrease (Figure 1A). Masson's trichrome staining was used to observe the liver structure and liver fibrosis (Figure 1B). CCl_4_ treatment caused the destruction of the normal structure, accompanied by the formation of a fibrous septum and the formation of a pseudo-lobe in the liver of mice. However, in CCl_4_-induced liver fibrosis mice with NOX4 intervention, disorder in the hepatic lobular structure, fibrous space, and collagen deposition in liver tissue was alleviated compared with CCl_4_-induced liver fibrosis mice. Hepatic lobular structure and fibrous septum in the liver of liver fibrosis mice with RhoA intervention also improved significantly. Quantitative analysis also showed a significant improvement in the fibrotic score and area (*P* < 0.05) (Figures 1C,D). Then, we performed serum tests to assess liver function (Figure 1E). ALT, AST and TBIL changes in mouse serum results indicated liver damage caused by CCl_4_ treatment but this was partly recovered by NOX4 or RhoA intervention.

#### High-Throughput 16S Sequencing of Mouse Feces

**Figure 2:**
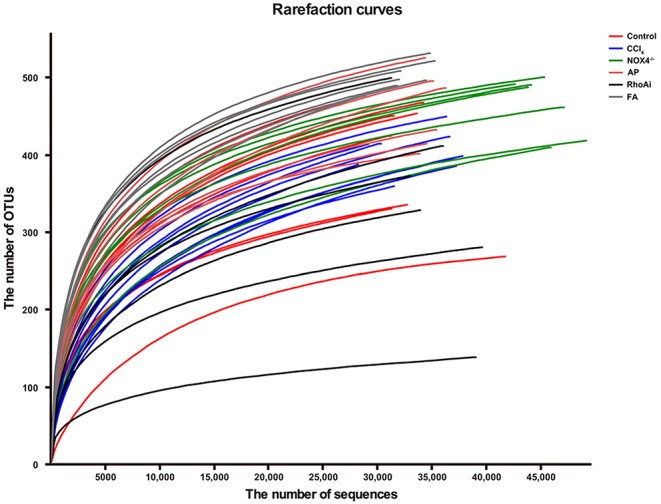
The rarefaction curve shows the number of bacterial OTUs at the indicated sequencing number in each sample. A random sampling sequence method was used to construct a rarefaction curve using the microbial diversity index of each sample at different sequencing depths to reflect the microbial diversity of each sample. The near-saturated rarefaction curve indicates that each sample was vastly microbially diverse. CCl_4_, carbon tetrachloride; NOX4^−/−^, NOX4 knockout; AP, apocynin; RhoAi, RhoA inhibitor; FA, fasudil.

The intestinal microbiota is an important part of the liver-gut axis, and the identification of changes in the microbiota is of great significance for the study of liver fibrosis. In recent years, 16S rRNA gene high-throughput sequencing technology has been increasingly used in the study of the intestine and disease relationships. 16S sequencing analysis can better reflect changes in bacterial diversity and composition compared to conventional culture methods. This study contains 1,744,366 high-quality sequences with a read length ≥200 bp. The proportion of the sequences with a length of 401–420 bp is 24%, 421–440 bp is 52%, and 441–460 bp is 24%. The rarefaction curve reaches the plateau stage, indicating sufficient sequencing (Figure 2). Moreover, based on the OTU analysis, total sequences were assigned to 1 domain, 1 kingdom, 17 phyla, 31 classes, 63 orders, 107 families, 215 genera, and 370 species from the feces of all the mice (Table 2).

**Table 2:** Bacterial communities in the feces of mice.

**Samples**	**Valid reads**	**Domains**	**Kingdoms**	**Phyla**	**Classes**	**Orders**	**Families**	**Genera**	**Species**
Control	32,993	1	1	14	26	55	92	169	269
CCl_4_	33,671	1	1	10	17	31	57	119	209
NOX4^−/−^	45,301	1	1	11	19	28	49	115	214
AP	34,755	1	1	11	19	29	49	124	228
RhoAi	35,365	1	1	10	19	30	54	111	190
FA	32,703	1	1	8	16	26	47	112	196

#### Effect of the Profibrotic Factors NOX4 and RhoA on the Di Versity of Bacteria in Liver Fibrosis Mice

**Figure 3:**
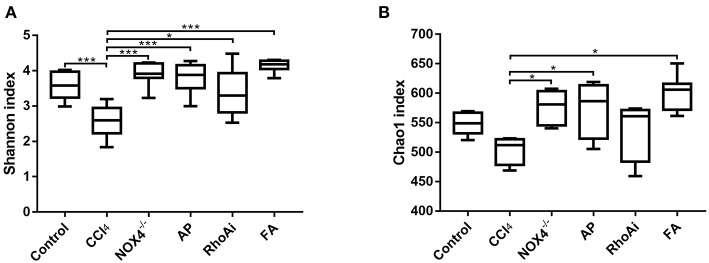
Analysis of the alpha diversity of microbiota in each sample. **(A)** The Shannon index is used to estimate the diversity of species of the microbiota in each sample. **(B)** The Chao1 index was used to estimate the number of species in the microbiota in each sample. The species diversity and number in the microbiota were estimated through a series of statistical analysis indexes, reflecting the alpha diversity of the microbiota. Alteration of NOX4 or RhoA expression can improve the alpha diversity of the microbiota in liver fibrosis mice. Data represent the mean ± SD of values for each group (*n* = 6). Student's *t*-test was used to analyze the differences between groups. *P < 0.05 and ***P < 0.001. CCl_4_, carbon tetrachloride; NOX4^−/−^, NOX4 knockout; AP, apocynin; RhoAi, RhoA inhibitor; FA, fasudil.

To better define the relationship between intestinal microbiota changes and the profibrotic factors NOX4 and RhoA, we studied the diversity and composition of intestinal microbiota in liver fibrosis mice. First, we tested the alpha diversity of the intestinal microbiota. The Shannon index was used to assess the diversity of the microbiota. The results suggest that the value of the Shannon index of the bacteria in liver fibrosis mice is significantly lower than that in normal mice. However, in the liver fibrosis mice with NOX4 or RhoA intervention, the decreased Shannon value increased (Figure 3A). Similarly, the Chao1 index, the estimated microbial abundance, was decreased in the CCl_4_ group compared with the control group, and there was a increase in the NOX4^−/−^, AP, RhoAi, and FA groups (Figure 3B), indicating that intestinal microbiota diversity and abundance decreased in liver fibrosis mice, and this decline was associated with NOX4 and RhoA.

#### Effect of Profibrotic Factors NOX4 and RhoA on the Bacterial Microbiota Composition of Liver Fibrosis Mice

**Figure 4:**
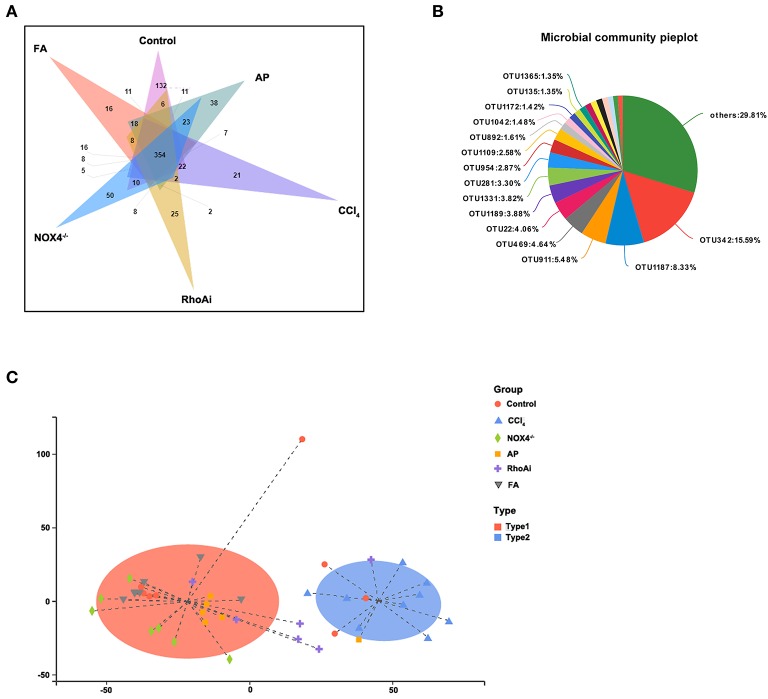
Analysis of microbiota structure in each group. **(A)** A Venn diagram is used to show the number of common and unique OTUs in different groups. **(B)** Composition ratio of the common OTUs between different groups. **(C)** Typing analysis of different groups of bacteria. The Jensen-Shannon distance (JSD) equidistance was calculated based on the relative abundance of the flora at the classification level, and PAM (partitioning around medoids) clustering was performed. The Calinski-Harabasz (CH) index was used to calculate the optimal clustering K value. Finally, these indexes were visualized. Alteration of NOX4 or RhoA expression can change the microbiota structure in liver fibrosis mice. CCl_4_, carbon tetrachloride; NOX4^−/−^, NOX4 knockout; AP, apocynin; RhoAi, RhoA inhibitor; FA, fasudil.

**Figure 5:**
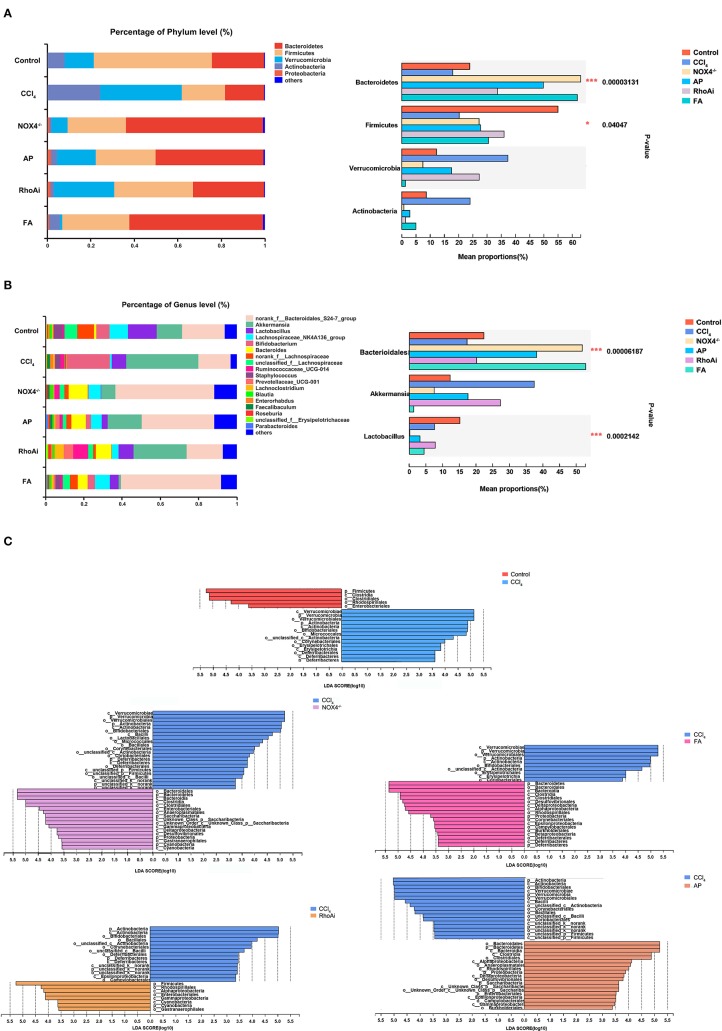
Composition analysis of microbiota in each group. **(A)** At the phylum level, the composition of the microbiota was analyzed in each group. **(B)** At the genus level, the composition of the microbiota was analyzed in each group. Based on the community abundance data, statistical methods were used to test hypotheses regarding the species between different groups of microbial communities, to evaluate the significance level of species abundance differences and to determine species with significant differences between groups. **(C)** Linear discriminant analysis (LDA) was used to analyze the taxon differences of the microbiota in all groups. LDA is a linear classifier that assigns objects to groups based on Mahalanobis distance from the object to the center of the group to estimate the impact of the abundance of each species on the effect of the difference. Microbiota composition changes during liver fibrosis and partial recovery after NOX4 and RhoA intervention. One-way ANOVA was used to analyze the differences between groups. *P < 0.05 and ***P < 0.001. CCl_4_, carbon tetrachloride; NOX4^−/−^, NOX4 knockout; AP, apocynin; RhoAi, RhoA inhibitor; FA, fasudil.

Disorders of the bacterial microbiota composition often occur in liver fibrosis. Next, we analyzed the composition of the microbiota of the animal model. To obtain an overall understanding of the composition of the sample, we counted the number of OTUs common and unique in the sample. The Venn plot shows that there are a total of 354 common species in all mice (Figure 4A). The OTU distribution of these common species is shown in Figure 4B. All sample populations were analyzed (Figure 4C). The results showed that most of the intestinal microbiota of the control, NOX4^−/−^, AP, RhoAi, and FA groups were divided into one type, while the that of CCl_4_ group was divided into another type, indicating that the intestinal microbiota of liver fibrosis mice tended to be normal after intervention with NOX4 or RhoA. Then, we studied the composition of the intestinal microbiota at the phylum level in each group (Figure 5A). Firmicutes, which include beneficial bacteria such as *Lactobacillus*, and Bacteroidetes showed a significant decrease in liver fibrosis in mice, but in the NOX4 or RhoA intervention group, Firmicutes and Bacteroidetes showed a increase compared with the CCl_4_ group. At the genus level, in liver fibrosis mice, *Lactobacillus* could participate in important body activities (Liu et al., 2004; Bajaj et al., 2014), showing a significant decrease. In the RhoAi group, *Lactobacillus* was slightly higher than in the CCl_4_ group. Bacteroidales and Lachnospiraceae decreased in liver fibrosis mice, and partial recovery occurred in the NOX4^−/−^, AP, RhoAi, and FA groups (Figure 5B). Moreover, Linear discriminant analysis (LDA) can detect the characteristics of significant abundance differences in the sample and find the microbiota with significant differences in abundance, which is used to reveal the characteristics of the components of each group (Figure 5C). The results showed that Firmicutes was the most enriched in the control and RhoAi groups, Verrucomicrobia, and Actinobacteria were enriched in the CCl_4_ group, and Bacteroidetes was significantly enriched in the NOX4^−/−^, AP and FA groups, indicating that intestinal microbiota are disordered during liver fibrosis, but when NOX4 or RhoA is inhibited, the disordered microbiota has a recovery, suggesting that the disorder is associated with profibrotic factors.

#### Relationship Between the Microbial Community and Environmental Factors

**Figure 6:**
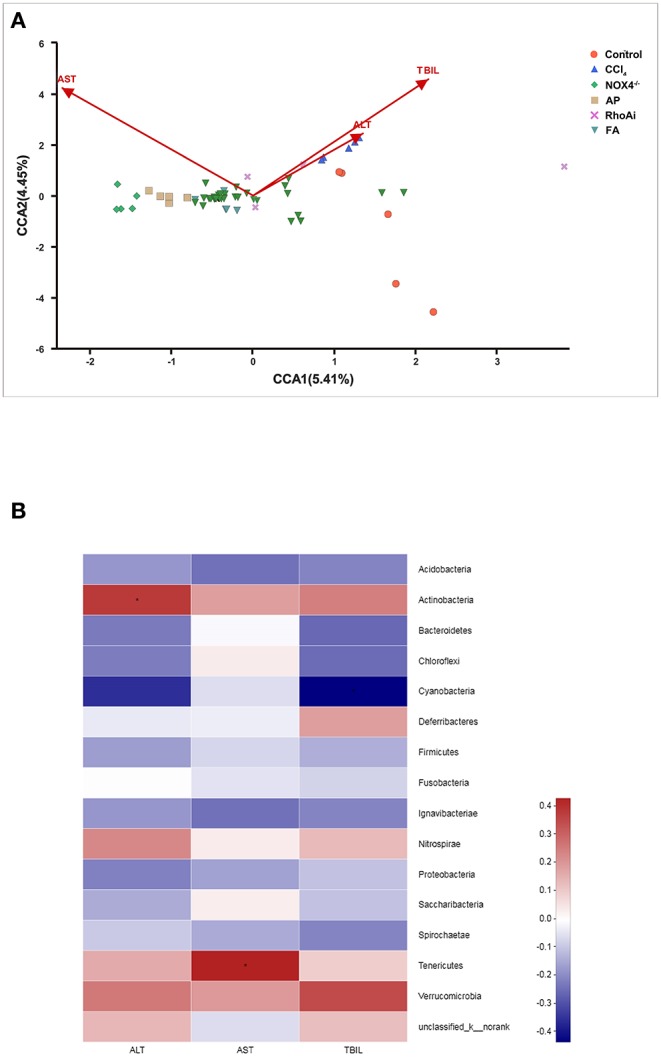
Correlation analysis between physiological indicators and microbiota. **(A)** A canonical correspondence analysis (CCA) ordination plot assessed the correlation between serum indicators and microbiota in all sample. CCA analysis combines correspondence analysis and multiple regression analysis, and each step of the calculation is performed with environmental factors, which is mainly used to reflect the relationship between the flora and environmental factors. **(B)** Heat mapping was used to assess the correlation between serum indicators and microbiota species in all samples. The X-axis and Y-axis are environmental factors and species, respectively. Correlation values were obtained through calculation and are displayed in different colors. The red and blue represent the positive and negative correlations, respectively. AST (*P* = 0.001) and TBIL (*P* = 0.002) were significantly related to the bacterial community structure. The Mann-Whitney rank sum test was used to test the correlation between the microbial community and environmental factors. *P < 0.05. CCl_4_, carbon tetrachloride; NOX4^−/−^, NOX4 knockout; AP, apocynin; RhoAi, RhoA inhibitor; FA, fasudil; ALT, alanine transaminase; AST, aspartate aminotransferase; TBIL, total bilirubin.

When liver fibrosis occurs, some biochemical factors of the body often appear abnormal (Mohamadnejad et al., 2006). We suspect that there may be a link between changes in these indicators and the structure of the microbiota. To explore the potential correlations between the bacterial community and the physiological factors in animal models, redundancy analysis (RDA) was used. RDA can compare the bacterial community to environmental variables based on biochemical parameters and assess the correlation between the two. We included the serum index for testing. As shown in Figure 6A, AST (*P* = 0.001) and TBIL (*P* = 0.002) significantly related to the bacterial community structure. We also analyzed the effects of different serum indices on the community composition of the microbiota. Correlation heatmap analysis results show that ALT is significantly positively correlated with Actinobacteria (*r* = 0.38, *P* = 0.04), and AST is significantly positively correlated with Tenericutes (*r* = 0.43, *p* = 0.02) (Figure 6B).

#### Prediction of the Function of Microbiota

**Figure 7:**
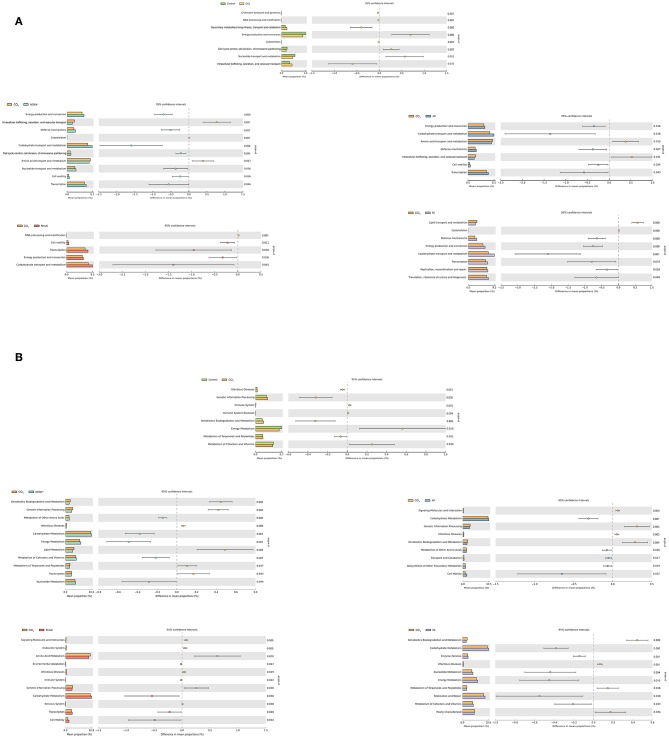
Predicting the function of the microbiota by querying the COG and KEGG databases. **(A)** Analysis of COG pathways for enrichment and difference between all groups. **(B)** Analysis of KEGG pathways for enrichment and difference between all groups. The OTU abundance table of the microbiota was normalized using PICRUSt software, and then the COG and KEGG OTU information was obtained through the Greengenes id corresponding to each OTU. In liver fibrosis mice with altered NOX4 or RhoA expression, the enrichment pathway caused by microbiota changes differed from that of liver fibrosis mice. The Mann-Whitney rank sum test was used to analyze the differences between groups. CCl_4_, carbon tetrachloride; NOX4^−/−^, NOX4 knockout; AP, apocynin; RhoAi, RhoA inhibitor; FA, fasudil.

To predict the changes in function corresponding to the changes in the microbiota in different groups of mice, we searched the COG and KEGG databases. We used PICRUSt software for analysis. The COG database mainly contains information about bacteria and archaea genomes (Chang and Ramasamy, 2018). In CCl_4_-induced liver fibrosis mice, the enriched COG terms are as follows: “Secondary metabolites biosynthesis, transport and catabolism,” “Cytoskeleton,” and “Intracellular trafficking, secretion, and vesicular transport.” In NOX4 knockout and AP treatment liver fibrosis mice, the enriched COG terms are “Energy production and conversion,” “Defense mechanisms,” “Carbohydrate transport and metabolism,” “Cell motility,” and “Transcription.” In addition, “Carbohydrate transport and metabolism,” “Energy production and conversion,” “Replication, recombination and repair,” and “Translation, ribosomal structure and biogenesis” are the enriched terms in RhoAi-inhibited or FA-treated liver fibrosis mice (Figure 7A).

Next, we compared the KEGG database pair to explore the molecular biosignalling pathways predicted by the microbiota change (Figure 7B). In the CCl_4_ group, the main metabolic pathways predicted are “Infectious Diseases,” “Genetic Information Processing,” “Xenobiotics Biodegradation and Metabolism,” and “Metabolism of Terpenoids and Polyketides.” Compared with the CCl_4_ group, the main metabolic pathways are “Carbohydrate Metabolism,” “Energy Metabolism,” “Metabolism of Cofactors and Vitamins,” and “Nucleotide Metabolism” in the NOX4^−/−^ group and “Carbohydrate Metabolism,” “Metabolism of Other Amino Acids,” “Transport and Catabolism,” and “Biosynthesis of Other Secondary Metabolites” in the AP group. In addition, in the RhoAi group, “Carbohydrate Metabolism,” “Transcription,” and “Cell Motility” are the enriched pathways. In the FA group, “Enzyme Families,” “Replication and Repair,” and “Carbohydrate Metabolism” are the main pathways associated with bacteria change. In animal models of liver fibrosis and NOX4 or RhoA intervention, functional changes in function predicted by changes in flora may indicate changes in life activity in the body. The results suggest that the changes in metabolic pathways caused by bacterial microbiota may be associated with disease.

## Discussion

In this study, we show that the intestinal microbiota are disordered during liver fibrosis. Based on high-throughput sequencing technology, we analyzed the diversity, abundance and functional prediction of intestinal microbiota to confirm the correlation between the disorder and the profibrotic factors NOX4 and RhoA.

In recent years, changes in the intestinal microbiota have been found in many chronic liver diseases, and this change is closely related to the development of liver disease (Leung et al., 2016; Ferrere et al., 2017; Marra and Svegliati-Baroni, 2018). However, few studies have focused on changes in intestinal microbiota during liver fibrosis. As an early pathological change in chronic liver disease, liver fibrosis can cause damage to liver tissue and interfere with the normal physiological functions of the liver (Lee et al., 2015). The metabolism of intrahepatic disorders affects intestinal homeostasis through the liver-gut axis (Visschers et al., 2013). Our previous study found that intestinal damage and intestinal barrier destruction occur in a CCl_4_-induced mouse liver fibrosis model. To this end, we analyzed the changes in the intestinal microbiota during liver fibrosis. Consistent with findings from cirrhosis studies, the present study showed that the diversity and abundance of the intestinal microbiota decreased significantly in liver fibrosis mice. In terms of microbiota composition, we found that the intestinal microbiota of the liver fibrosis mice and the control mice belonged to two types. The abundances of the phylum Firmicutes and genus *Lactobacillus* showed decreases in CCl_4_-induced mice. Regarding functional prediction, this change in the microbiota may indicate the occurrence of adverse metabolic pathways, suggesting that the intestinal microbiota is disordered during liver fibrosis. Interestingly, we did not observe a significant change in *Escherichia coli*. The possible cause of this observation is the early stage of the liver fibrosis, at which point it is too early to cause dramatic changes in all bacteria.

A notable finding in this study is that changes in the gut microbiota during liver fibrosis may be associated with profibrotic factors. NOX4 and RhoA are important profibrotic factors and are closely related to each other. We have demonstrated that Rac1 is involved in NOX activation and that NOX4/ROS can also induce HSC activation by activating the RhoA/ROCK1 signaling pathway, thereby promoting liver fibrosis. Therefore, we speculate whether the disorder of intestinal microbiota in liver fibrosis is related to NOX4 and RhoA. We constructed animal models of NOX4 or RhoA intervention by gene knockout, AAV injection and bioinhibitor injection. The results showed that in the liver fibrosis mice treated with NOX4 or RhoA, the disordered intestinal microbiota showed different degrees of improvement. This finding confirms that liver fibrosis dysbacteriosis has a correlation with NOX4 and RhoA activity and that interference with NOX4 and RhoA function can partially correct the disorder of the intestinal microbiota, which may be a potential mechanism for NOX4 and RhoA to promote the development of liver fibrosis. However, there are still some shortcomings in this study. The specific mechanism between profibrotic factors and dysbacteriosis and the interaction between NOX4 and RhoA in dysbacteriosis are not clear. This additional information requires many further experiments.

With the gradual deepening of the understanding of the intestinal microbiota, an increasing number of scholars are aware that the intestinal microbiota may be closely related to the disease. The liver acts as an organ closely related to the intestinal microbiota, and changes in the intestinal microbiota during liver fibrosis need to be closely monitored. Disordered intestinal microbiota may cause an intrahepatic immune inflammatory response through the liver-gut axis (Seki and Brenner, 2008), which may aggravate the progression of liver fibrosis to end-stage liver disease. The identification of changes in the microbiota helps us gain a better understanding of the underlying pathogenesis of liver fibrosis. In addition, by studying the molecular and signaling pathways associated with intestinal microbiota changes, new ideas for targeting the intestinal microbiota for the treatment of liver fibrosis can be proposed.

In conclusion, our study found that there is a disorder in the intestinal microbiota during liver fibrosis, and this disorder may be related to profibrotic factors NOX4 and RhoA. The current research provides new insight into the potential mechanism of liver fibrosis development. Although the molecular mechanism of liver fibrosis affecting intestinal microbiota has not yet been clarified, this study provides theoretical support for a better understanding of the underlying mechanisms of liver fibrosis development and the development of diagnostic and therapeutic liver fibrosis based on intestinal microbiota.

## Data Availability Statement

All datasets generated for this study are included in the article/Supplementary Material.

## Ethics Statement

The animal study was reviewed and approved by Animal Care and Use Committee of Nanchang University.

## Author Contributions

SW and YN designed and analysed the data. YZ and CH wrote the manuscript. XZ critically revised the manuscript.

### Conflict of Interest

The authors declare that the research was conducted in the absence of any commercial or financial relationships that could be construed as a potential conflict of interest.
