# Supplementary material for: Gut Microbial Dysbiosis Is Associated With Profibrotic Factors in Liver Fibrosis Mice
Source: Front Cell Infect Microbiol. 2020 Jan 31;10:18. doi: 10.3389/fcimb.2020.00018 (PMC7004962; doi:10.3389/fcimb.2020.00018)
Supplement: Supplementary file 1 [file Table_1.DOCX]

**TABLE 1 Primer sequences for RT-qPCR**

| PCR gene name | Forward primer (5′–3′) | Reverse primer (5′–3′) |
| --- | --- | --- |
| RhoA | AGCTTGTGGTAAGACATGCTTG | GTGTCCCATAAAGCCAACTCTAC |
| GAPDH | ATGGGTGTGAACCACGAGA | CAGGGATGATGTTCTGGGCA |
