# Supplementary material for: Gut Microbial Dysbiosis Is Associated With Profibrotic Factors in Liver Fibrosis Mice
Source: Front Cell Infect Microbiol. 2020 Jan 31;10:18. doi: 10.3389/fcimb.2020.00018 (PMC7004962; doi:10.3389/fcimb.2020.00018)
Supplement: Supplementary file 2 [file Data_Sheet_1.docx]

**Supplementary Figure 1** Verification of inhibited RhoA gene expression in mice by administration of an adeno-associated virus (AAV) virus. The hepatic mRNA levels of RhoA were measured by real-time RT-PCR. In mice injected with the AAV virus, RhoA expression in the liver was significantly decreased. Data represent the mean ± SD of values in each group (n=3). Student's t test was used to analyse the differences between groups. ***P < 0.001.

**Supplementary Table 1 Primer sequences for RT-qPCR**
