# Supplementary figures and images for: Gut Microbial Dysbiosis Is Associated With Profibrotic Factors in Liver Fibrosis Mice
Source: Front Cell Infect Microbiol. 2020 Jan 31;10:18. doi: 10.3389/fcimb.2020.00018 (PMC7004962; doi:10.3389/fcimb.2020.00018)

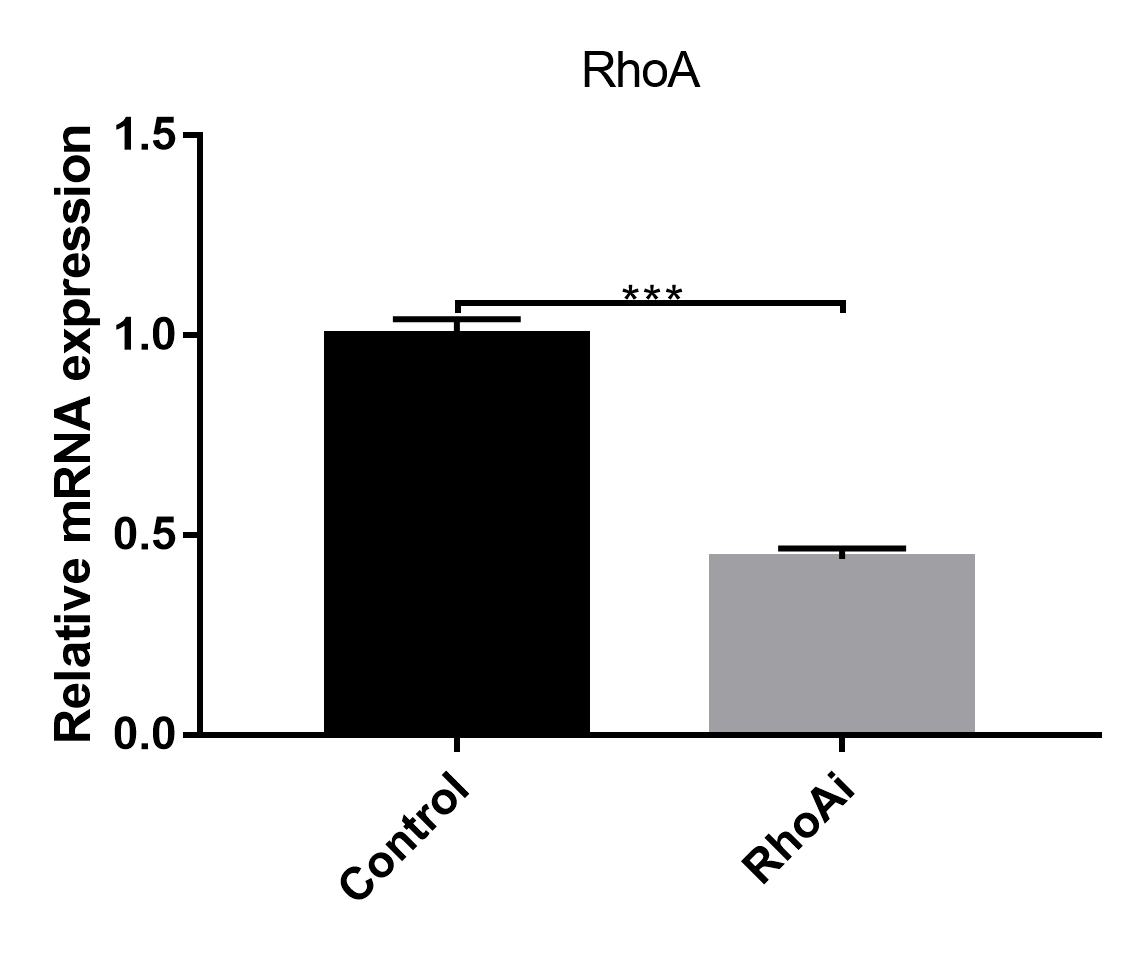

Supplement: Supplementary file 3 [file Image_1.TIF]
